# Biomagnification and Temporal Trends of New and Emerging Dechloranes and Related Transformation Products in Baltic Sea Biota

**DOI:** 10.1021/acs.estlett.2c00171

**Published:** 2022-04-13

**Authors:** Peter Haglund, Andriy Rebryk

**Affiliations:** Department of Chemistry, Umeå University, 901 87 Umeå, Sweden

**Keywords:** Dechloranes, emerging contaminants, transformation products, suspect screening, biomagnification, temporal trends, top predators

## Abstract

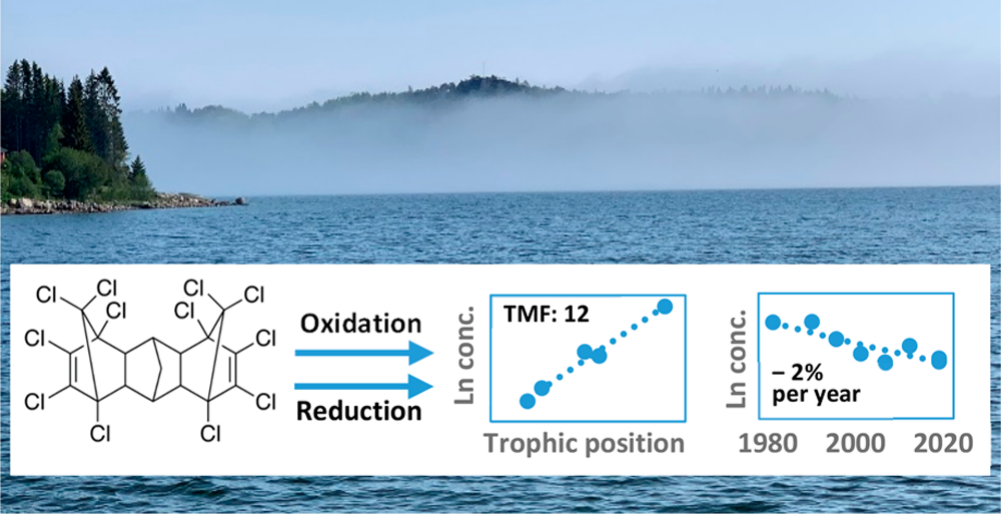

To enhance knowledge of the environmental distribution and temporal trends of dechloranes and their transformation products (TPs) we performed suspect screening of Baltic Sea biota (eelpout, herring, harbor porpoise, guillemot and white-tailed sea eagle). Evaluation of new and “digitally frozen” gas chromatography/high-resolution mass spectrometry data revealed 31 compounds: five dechloranes (Dechlorane [Mirex], Dechlorane 602, Dechlorane 603, and *syn*-/*anti*-Dechlorane Plus [DP]), three isomers, and 23 TPs. Six new Dechlorane 603 TPs and two new DP TPs were detected, including one hydroxy-TP. Some TPs occurred at much higher concentrations than the parent compounds (e.g., Dechlorane 603 TPs were >10-fold more abundant than their parent). Concentrations of contaminants in the most contaminated species (white-tailed sea eagle) changed little over the period 1965–2017. Slow declines were detected for most compounds (median, 2% per year), although concentrations of DP and DP-TPs increased by 1% per year. Ten contaminants biomagnify, and the trophic magnification factors for TPs of Mirex, Dechlorane 602 and Dechlorane 603 (8.2 to 17.8) were similar to the parent compounds (6.6 to 12.4) and higher than that of DP (2.4, nonsignificant). The results are discussed in relation to the current review of DP for potential listing under the Stockholm Convention on POPs.

## Introduction

Persistent organic pollutants (POPs) have been recognized as environmental hazards and are regulated through the Stockholm Convention on POPs.^1^ Early discoveries of POPs generally involved bottom-up approaches and an element of chance. First, the substance was identified in the environment, and then its origin was investigated. For instance, Jensen found enormous quantities of unknown substances when analyzing DDT (dichloro-diphenyl-trichloroethane), which were later identified as PCBs.^2^ Later, top-down approaches were developed involving ‘*in-silico* screening’ of substance inventories to find compounds with properties similar to those of the regulated POPs.^3,4^

Recent advances in analytical instruments, particularly for liquid chromatography (LC) coupled to high-resolution mass spectrometry (HRMS), gas chromatography (GC) coupled to HRMS or low-resolution MS, and comprehensive two-dimensional GC or LC (GC × GC and LC × LC) with MS detection, have led to a paradigm shift in POP discovery. These instruments enable the capture of signatures (mass spectra) of all constituents of a sample that reach the detector, opening avenues for hypothesis-driven screening of new POPs using so-called suspect and nontarget screening.^5,6^

In 2021, a biomagnification factor (BMF)-driven nontarget screening attempt was published.^7^ This showed that diverse contaminants (including legacy POPs, halogenated natural products, polycyclic aromatic hydrocarbons (PAHs), and the novel flame retardant Dechlorane 602) biomagnify in tissues of Baltic Sea species. Data from such studies may be stored in digital archives, and the availability of “digitally frozen” samples may reduce the need to use precious frozen tissue material from Environmental Specimen Banks (ESBs) for retrospective analysis.^8,9^

The study presented here was triggered by the discovery of Dechlorane 602 (Dec602) in Baltic Sea biota and the realization that it biomagnifies (with BMFs of 3.0 to 20) in several top predators.^7^ A literature search revealed the occurrence of additional dechloranes in aquatic biota including Dechlorane (Mirex), Dechlorane 603 (Dec603), Dechlorane 604 (Dec604), *syn*-/*anti*-Dechlorane Plus (syn-/anti-DP), and Chlordene Plus (CP).^10^ It also showed that these compounds undergo dechlorination (forming mono- or dihydro-analogues)^11−15^ and oxidation (forming carbonyls).^13^ This prompted suspect screening of dechloranes, hydrodechloranes, and carbonyl and hydroxyl transformation products (TPs) of dechloranes and hydrodechloranes. Screening of samples collected in recent years revealed the presence of several new and emerging dechloranes in Baltic Sea biota. Digitally frozen samples were then used to investigate their temporal trends and trophic biomagnification.

## Materials and Methods

### Samples

All samples used in this study were collected from the Swedish part of the Baltic Proper (Supporting Information, Table S1) by the Swedish Museum of Natural History (SMNH) and stored in their ESB, with permission from Stockholm regional ethical review board. The following tissue samples were used: eelpout (*Zoarces viviparus*) muscle (1995–2017, *n* = 9), herring (*Clupea harengus*) muscle (1986–2018, *n* = 12), harbor porpoise (*Phocoena phocoena*, hereafter porpoise) blubber (1988–2019, *n* = 9), guillemot (*Uria aalge*) eggs (1986–2019, *n* = 12), and white-tailed sea eagle (*Haliaeetus albicilla*, hereafter eagle) muscle (1965–2017, *n* = 8). Subsamples were pooled to reduce biological variance (Table S1). Because of shortage of material, single samples of eagle (collected 1965) and porpoise (2001 and 2003) tissue were used. The Department of Environmental Science and Analytical Chemistry of Stockholm University, Sweden, provided archived (−18 °C), samples of guillemot lipids, left-over from previous analyses of tissues of individuals obtained from the SMNH ESB, which were used to prepare pooled samples. Guillemot lipids were extracted following published protocols.^16^ All other pooled samples were prepared from tissue stored (−25 °C) in the ESB.

### Extraction, Clean-up and Analysis

Samples were extracted with acetone:*n*-hexane and *n*-hexane:diethyl ether, and lipid weights (l.w.) were determined gravimetrically.^7^ Bulk lipids were removed by high-resolution gel permeation chromatography (HR-GPC), as described in Table S2. Contaminant fractions were collected, concentrated, and subjected to a second round of HR-GPC. Samples were further fractionated using a Florisil column (Table S2),^7^ and the first three fractions (*n*-hexane, 15% dichloromethane in *n*-hexane, and 50% dichloromethane in *n*-hexane) were retained. Volumetric standard (^13^C_12_–CB-188; 3 ng) was added to each fraction, and their volumes were reduced to 0.3 mL. They were then subjected to GC-HRMS using an Agilent 7250 (Santa Clara, CA, USA) system operating in electron ionization (EI) or methane electron capture chemical ionization (ECNI) mode, as previously described.^7^ EI was used for Mirex and its TPs, and ECNI for the other compounds (Table S3). Suspect screening was performed using extracted ion chromatograms of C_5_Cl_6_ fragment ions (*m*/*z* 271.8096) of Mirex and monohydro-Mirex, molecular ions of Dec602, Dec603, Dec604, DP, and CP, and molecular ions of the associated mono/di/trihydro-TPs, mono/dicarbonyl-TPs, and monohydroxy-TPs. Dechloranes were quantified (ng/g l.w.) in the most recent sample of each species (by reanalysis of archived (−18 °C) purified extracts from a previous study)^33^ utilizing the lipid weight normalized peak area ratio (AR l.w.) of the reference standard (AccuStandard, New Haven, CT, USA) and the volumetric standard. TPs were quantified by comparing their peak areas with those of the parent compounds, assuming that they have identical instrument responses. Subsequently, earlier samples were quantified using “digitally frozen” samples (digitally archived GC full-spectrum high-resolution accurate mass MS data), with the most recent sample as reference, using the following equation: concentration in year Y = concentration in reference year × AR l.w. in year Y/AR l.w. in reference year. When an analyte split between Florisil fractions, its concentration in each fraction was quantified separately and the results were combined.

### Temporal Trend Calculations

Annual changes in contaminant concentrations were obtained by linear regression of the natural logarithms of the concentrations and sample collection year. This results in regression lines representing percentage annual changes in their concentrations. The log-transformed data were visually checked for normality by plotting frequency distributions (histograms) of the data and comparing it to a normal distribution curve. The data appeared to be normally distributed.

### Trophic Magnification Factor Calculations

Using stable nitrogen isotope-ratio measurements at the UC Davis Stable Isotope Facility (Davis, CA, USA) trophic levels (TLs) were calculated following published procedures^17^ and blue mussels as the reference species (average δ^15^N, 7.6; assigned TL, 2). Average TLs of the studied eelpout, herring, guillemot, porpoise, and eagle were 2.88, 2.91, 3.47, 3.59, and 4.25, respectively. Trophic magnification factors (TMFs) were calculated for the contaminants as the natural exponential function of b, where b is the slope of the linear regression between the natural logarithm of their concentration against the TL (TMF = e^b^, where b = slope). To improve the TMFs’ robustness, only data for samples collected between 2010 and 2020 were included.

## Results and Discussion

### Identification

In total, 31 dechloranes and dechlorane-related compounds were identified or tentatively identified (Table 1). Dechlorane (Mirex), Dec602, Dec603, *syn*-DP, and *anti*-DP were identified using standards. Ten compounds were assigned probable structures using published GC retention and MS data, that is, Level 2 identification confidence according to the scheme of Schymanski et al.^18^ The remaining compounds were tentatively identified (Level 3 confidence)^18^ by comparing their GC elution order and MS data to those of the parent compounds and established TPs. The identification confidence levels for all detected compounds are summarized in Table 1, and the proposed structures for the detected compounds are compiled in Figure S1.

**Table 1:** Concentrations (ng/g Lipids), Abbreviations, Florisil Fraction(s), Identification Confidence Levels (ID conf.),^18^ and Mass Accuracies (ppm; Experimental Mass – Theoretical Mass) of Dechloranes and Related Transformation Products Detected in Eelpout, Herring, Harbor Porpoise, Guillemot, and White-Tailed Sea Eagle from the Baltic Sea

compound	abbreviation	Florisil Fr.	ID conf.	ppm	eelpout (*n* = 9)	herring (*n* = 12)	porpoise (*n* = 9)	guillemot (*n* = 12)	eagle (*n* = 8)
Dechlorane (Mirex)	Mirex	1	1	1.1	0.40	0.33	6.7	13	547
Photomirex (8H-mirex)	Photomirex	1	2	0.4	0.21	0.16	2.6	2.4	153
10H-mirex	10H-mirex	1	2	–1.5	0.012	0.016	0.63	0.14	15
Dechlorane 602	Dec602	2 (1)	1	1.1	0.19	0.10	1.6	5.3	53
Dechlorane 602, isomer #1	Dec602, isomer #1	2	3	–2.0	<0.004	<0.004	<0.01	<0.01	5.2
Dechlorane 602, isomer #2	Dec602, isomer #2	2	3	1.8	<0.004	<0.004	<0.01	<0.01	0.80
Monohydro Dechlorane 602 #1	Hydro-Dec602 #1	2	3	–2.8	<0.004	<0.004	<0.01	0.23	2.1
Monohydro Dechlorane 602 #2	Hydro-Dec602 #2	2	3	0.3	<0.004	<0.004	<0.01	<0.01	0.86
11H-α-Dechlorane 602	11H-α-Dec602	2	2	4.3	0.016	0.012	<0.01	<0.01	3.7
Monohydro Dechlorane 602 #4	Hydro-Dec602 #4	2	3	2.3	<0.004	<0.004	<0.01	<0.01	2.7
11H-β-Dechlorane 602	11H-β-Dec602	2	2	3.1	0.006	0.006	0.62	1.1	18
10,11-dihydro-Dechlorane 602 (α)	10,11H-α-Dec602	2	2	–4.4	<0.003	0.003	<0.01	<0.01	0.66
10,11-dihydro-Dechlorane 602 (γ)	10,11H-γ-Dec602	2 (3)	2	–0.6	0.004	0.007	0.32	0.53	8.1
Dechlorane 603 (Dec603)	Dec603	2 (1)	1	0	0.001	0.003	0.039	0.057	3.5
Dechlorane 603, isomer	Dec603, isomer	2 (3)	3	1.4	<0.001	<0.002	0.010	0.008	3.4
Monohydro Dechlorane 603	U1	2	2	2.0	0.004	0.002	0.42	0.39	21
Dihydro Dechlorane 603	Dihydro-Dec603	3 (2)	3	–3.0	<0.002	<0.002	0.014	0.18	0.41
Monohydro Dec603, carbonyl-	U2	3	2	–1.9	0.005	0.003	0.18	0.17	5.3
Dihydro Dec603, carbonyl- #1	Monohydro-U2 #1	3	3	–0.3	<0.002	<0.002	0.034	0.022	0.71
Dihydro Dec603, carbonyl- #2	Monohydro-U2 #2	3	3	–2.9	<0.002	<0.002	0.17	0.010	3.4
Dihydro Dec603, carbonyl- #3	Monohydro-U2 #3	3	3	–0.7	0.004	<0.002	0.41	0.016	10
Trihydro Dec603, dicarboxy- #1		3	3	0.7	<0.002	<0.002	0.036	0.034	0.81
Trihydro Dec603, dicarboxy- #2		3	3	–3.9	<0.002	<0.002	0.051	0.059	1.4
Monohydro Dec603, hydroxy-	Hydroxy-U1	3	3	–4.9	<0.002	<0.002	<0.005	<0.005	0.067
Dechlorane Plus, syn-	syn-DP	2 (1)	1	2.6	0.12	0.047	0.012	0.11	2.4
Dechlorane Plus, anti-	anti-DP	2 (1)	1	3.1	0.28	0.10	0.037	0.33	4.5
Monohydro Dechlorane Plus, syn-	syn-Cl_11_-DP	2	2	3.9	<0.002	<0.002	<0.008	<0.009	2.9
Monohydro Dechlorane Plus, anti-	anti-Cl_11_-DP	2	2	4.5	<0.002	<0.002	<0.008	0.042	0.86
Dihydro Dechlorane Plus, anti-	anti-Cl_10_-DP	2	2	–1.4	<0.002	<0.002	<0.008	<0.009	0.023
Dechlorane Plus, carbonyl- #1	DP, carbonyl #1	3	3	–3.6	<0.002	<0.002	0.009	0.12	0.55
Dechlorane Plus, carbonyl- #2	DP, carbonyl #2	3	3	1.3	0.003	<0.002	0.010	0.11	0.57

Photomirex (8H-mirex) and 10H-mirex were tentatively identified using published GC retention and MS data.^11^ Nine Dec602-related compounds were detected, and seven were tentatively identified as five monohydro-Dec602 (including 11H-α-Dec602 and 11H-β-Dec602) and two dihydro-Dec602 (10,11H-α-Dec602 and 10,11H-γ-Dec602) using data published in another study.^12^ In addition, two isomers of Dec602 (with the same molecular formula) were detected, which may or may not be Dec602 positional isomers. Ten Dec603-related compounds were detected, most of them for the first time. Only two Dec603 TPs had previously been reported, a monohydro-Dec603 and its carbonyl, in a study where they were denoted U1 and U2.^13^ The remaining Dec603-related compounds were assigned as a Dec603 isomer, a dihydro-Dec603 (possibly a monohydro-TP of U1), three dihydro-Dec603 carbonyls (possibly monohydro-TPs of U2), two trihydro-Dec603 dicarbonyls, and one hydroxyl-Dec603. Liu et al. suggested (but did not confirm) that the latter may be an intermediate in the transformation of Dec603 to U2.^13^ They suggested that Dec603 is dechlorinated to monohydro-Dec603 (U1), hydroxylated to hydroxyl-Dec603, and further oxidized to U2. It is plausible that trihydro-Dec603 dicarbonyls are formed by further dechlorination and oxidation of monohydro-U2. Finally, five DP transformation products were detected, of which three were tentatively identified as monohydro *syn*-DP and *anti*-DP (*syn*-/*anti*-Cl_11_-DP) and dihydro-*anti*-DP (*anti*-Cl_10_-DP) using published data.^15^ The remaining two were assigned as DP-carbonyls.

**Figure 1:**
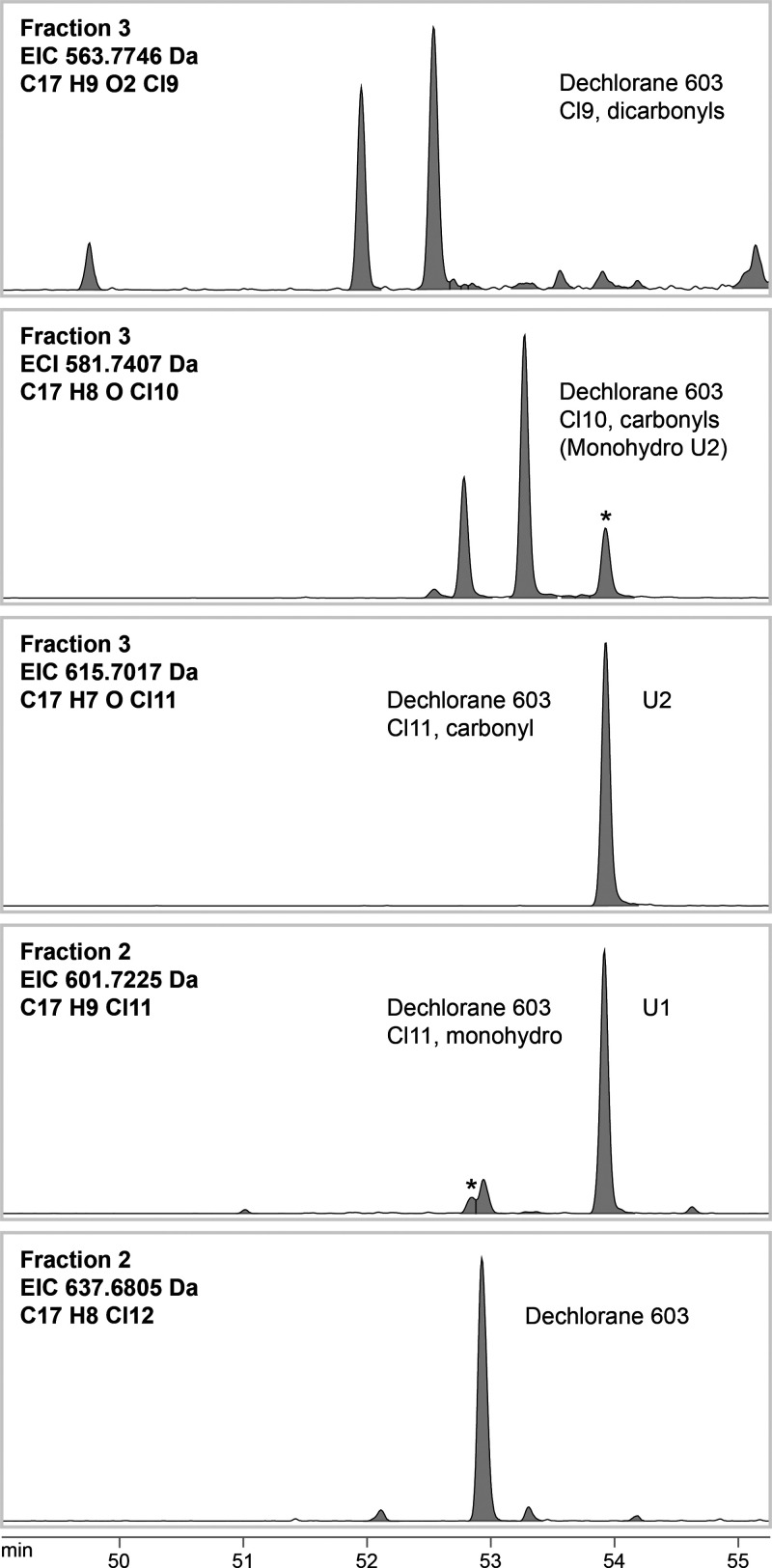
Extracted ion chromatograms (EICs, 50–55 min) from gas chromatography/high-resolution mass spectrometry analysis of Dechlorane 603 and related transformation products in Florisil Fractions 2 and 3 of a muscle sample of white-tailed sea eagle from the Baltic Sea. The asterisks indicate a fragment peak of a more chlorinated analogue (displayed in the panel below). U1 and U2 refer to a monohydro-Dechlorane 603 isomer and its carbonyl oxidation product identified in a previous study.^13^

Recorded masses of the analytes matched the theoretical masses well (within 5 ppm, Table 1). The molecular formulae, retention times, linear retention indices, quantification ions, and percent distributions among Florisil fractions are given in Table S3, and the spectra of new and emerging TPs of Dec603 and DP are shown in Figure S2. The GC elution order of previously reported TPs is consistent with orders in cited references,^11−13,15^ and the elution order of new TPs is consistent with expectations, for example, the elution order of Dec603, monohydro-Dec603, and dihydro-Dec603 (Figure 1) is similar to that of their Dec602 analogues.^12^ The distributions of dechloranes and TPs among Florisil fractions are also consistent with expectations (eluting in order of increasing polarity). Mirex and monohydromirex elute in Fraction 1, dechloranes in Fractions 1 and 2, monohydro-dechloranes in Fraction 2, dihydro-dechloranes in Fractions 2 and 3, and carbonyl/hydroxy-TPs in Fraction 3 (Table S3). All reported compounds were absent in procedural blanks. Dec604, CP, and their TPs^14^ were also sought but not found.

It is not possible to assign exact structures to the new Dechlorane TPs only using GC-HRMS information. Their spectra are lean in ions that can provide structural information. They are dominated by molecular ions, fragment ions formed by successive losses of chlorine, and sometimes pentachlorocyclopentadiene (C_5_Cl_5_) and chlorine ions (Figure S2). The molecular ion masses and isotope distribution patterns could nevertheless be used to verify that the assigned number of chlorines and molecular formula were correct. In addition, it has been shown that replacement of a geminal chlorine at a methylene bridge carbon atom by hydrogen is a common route of dehalogenation in the environment for organochlorine pesticides (e.g., Mirex, toxaphene, and dieldrin) and other chlorinated compounds (e.g., dechloranes) produced using Diels–Alder cycloaddition reactions involving hexachlorocyclopentadiene.^19^ Accordingly, all of the previously reported TPs are missing one (hydromirex, 11H-Dechlorane 602, U1, U2, syn/anti-Cl_11_-DP) or two (10,11-dihydro-Dechlorane 602, and anti-Cl_10_-DP) of the parent compound’s geminal chlorines. It is therefore postulated that the new Dec603 TPs also have lost geminal chlorines from the two methylene-bridge carbon atoms (Figure S1). In dihydro-TPs, it is expected that one chlorine substituent has been lost from each methylene-bridge carbon atom.

### Tissue Concentrations

Concentrations of all analytes are shown in Table 1. The data for the parent compounds are quantitative, and those of the TPs are semiquantitative. The levels were lower (ca. 10-fold) in fish, and higher (>10-fold) in eagle, than in porpoise and guillemot. Twelve compounds were detected in all samples: Mirex, photomirex, 10H-mirex, Dec602, 11H-α-Dec602, 11H-β-Dec602, 10,11H-γ-Dec602, Dec603, monohydro-Dec603 (U1), monohydro-Dec603-carbonyl (U2), and *syn*-/*anti*-DP. The eagle data can be used to exemplify the relative abundance of the different detected compounds in the samples. Dechlorane and photomirex occurred at concentrations above 100 ng/g lipids, 10H-mirex, Dec602, 11H-β-Dec602, U1, and one monohydro-U2 at 10–100 ng/g lipids, and two monohydro-Dec602, one Dec 602 isomer, 11H-α-Dec602, 10,11H-γ-Dec602, Dec603, Dec603-isomer, U2, one monohydro-U2, one trihydro-Dec603-dicarbonyl, *syn*/*anti*-DP, and *syn*-C_11_-DP at 1–10 ng/g lipids. The remaining compounds occurred at concentrations between 0.023 and 0.86 ng/g lipids. Notably, in the three top predators there were substantial concentrations of dechlorane TPs, relative to their parent compounds; ΣTP concentrations were slightly below (20–79% for Mirex and Dec602 TPs), similar to (76–141% for *syn*-/*anti*-DP TPs), or much higher than (1400–3300% for Dec603 TPs) the respective parent.

Direct comparison with results of other studies is difficult as there are few available measurements in Baltic biota. However, concentrations of DP in the region have been reported^20^ including *syn*-DP and *anti*-DP concentrations (ng/g lipids) that agree well with those presented here: 0.035 and 0.070 versus 0.047 and 0.10 in herring muscle; <0.57 and <0.14 versus 0.12 and 0.28 in eelpout muscle; 0.040 and 0.074 versus 0.012 and 0.037 in porpoise blubber; 0.13 and 0.40 versus 0.11 and 0.33 in guillemot egg; and 3.7 and 7.8 versus 2.4 and 4.5 in eagle muscle.

### Temporal Trends

Significant temporal trends (*p* < 0.05; Table 2) were detected for 16 compounds in eagle, 12 in guillemot, seven in porpoise, and one (Dec602) in herring. Decreasing trends were observed for Mirex, Dec602, Dec603, and their TPs, ranging between −0.4% and −3.8% per year. Decreases exceeding 1% per year were detected for Mirex (in guillemot, porpoise, eagle), photomirex (porpoise, eagle), Dec602 (guillemot, eagle), 11H-β-Dec602 and 10,11H-γ-Dec602 (guillemot), and Dec603 and TPs (eagle). In contrast, slowly increasing concentrations (ca. 1% per year) of *syn*-/*anti*-DP and *syn*-/*anti*-C_11_-DP in eagle were detected. A nonsignificant increase of 3.3% per year in ΣDP concentrations in Greenland peregrine falcon eggs (1986–2014) was also previously detected.^21^

**Table 2:** Annual Change and Linear Regression *p*-Values for Dechloranes and Related Transformation Products in Herring, Guillemot, Harbor Porpoise, and White-Tailed Sea Eagle Samples from the Baltic Sea

	herring (1986–2018) (*n* = 12)		guillemot (1986–2019) (*n* = 12)		porpoise (1988–2019) (*n* = 9)		eagle (1965–2017) (*n* = 8)	
compound	annual change	*p*-value	AC	*p*-value	AC	*p*-value	AC	*p*-value
Dechlorane (Mirex)			–2.1%	0.001	–2.5%	0.01	–1.8%	0.006
Photomirex (8H-mirex)			–1.4%	0.005	–1.4%	0.04		
10H-mirex			–0.5%	0.006			–0.7%	0.002
Dechlorane 602 (Dec602)	–0.5%	0.03	–2.4%	<0.001			–1.0%	0.04
Monohydro Dechlorane 602 (#1)			–0.6%	0.007				
Monohydro Dechlorane 602 (11H-α)			–0.6%	0.001				
Monohydro Dechlorane 602 (11H-β)			–1.4%	<0.001	–0.6%	0.05		
Dihydro Dechlorane 602 (10,11-γ-H)			–1.6%	0.001				
Dechlorane 603 (Dec603)			–0.4%	0.0006			–2.1%	0.03
Dechlorane 603, isomer							–2.0%	0.002
Monohydro Dechlorane 603 (U1)			–0.5%	0.003	–0.9%	0.02		
Dihydro Dechlorane 603			–0.5%	<0.001			–1.7%	0.002
Monohydro Dechlorane 603, carbonyl- (U2)					–0.8%	0.01	–3.5%	0.003
Dihydro Dechlorane 603, carbonyl- #1							–1.4%	0.04
Dihydro Dechlorane 603, carbonyl- #2					–0.6%	0.02	–3.8%	0.003
Dihydro Dechlorane 603, carbonyl- #3					–0.7%	0.01	–3.6%	0.007
Trihydro Dec603, dicarbonyl- #1							–2.5%	0.004
Monohydro Dechlorane 603, hydroxy-							–2.0%	<0.001
syn-Dechlorane Plus (syn-DP)							0.9%	<0.001
anti-Dechlorane Plus (anti-DP)			–0.5%	0.003			0.7%	0.02
syn-Cl_11_-Dechlorane Plus							1.3%	<0.001
anti-Cl_11_-Dechlorane Plus							0.7%	0.01

### Biomagnification

**Figure 2:**
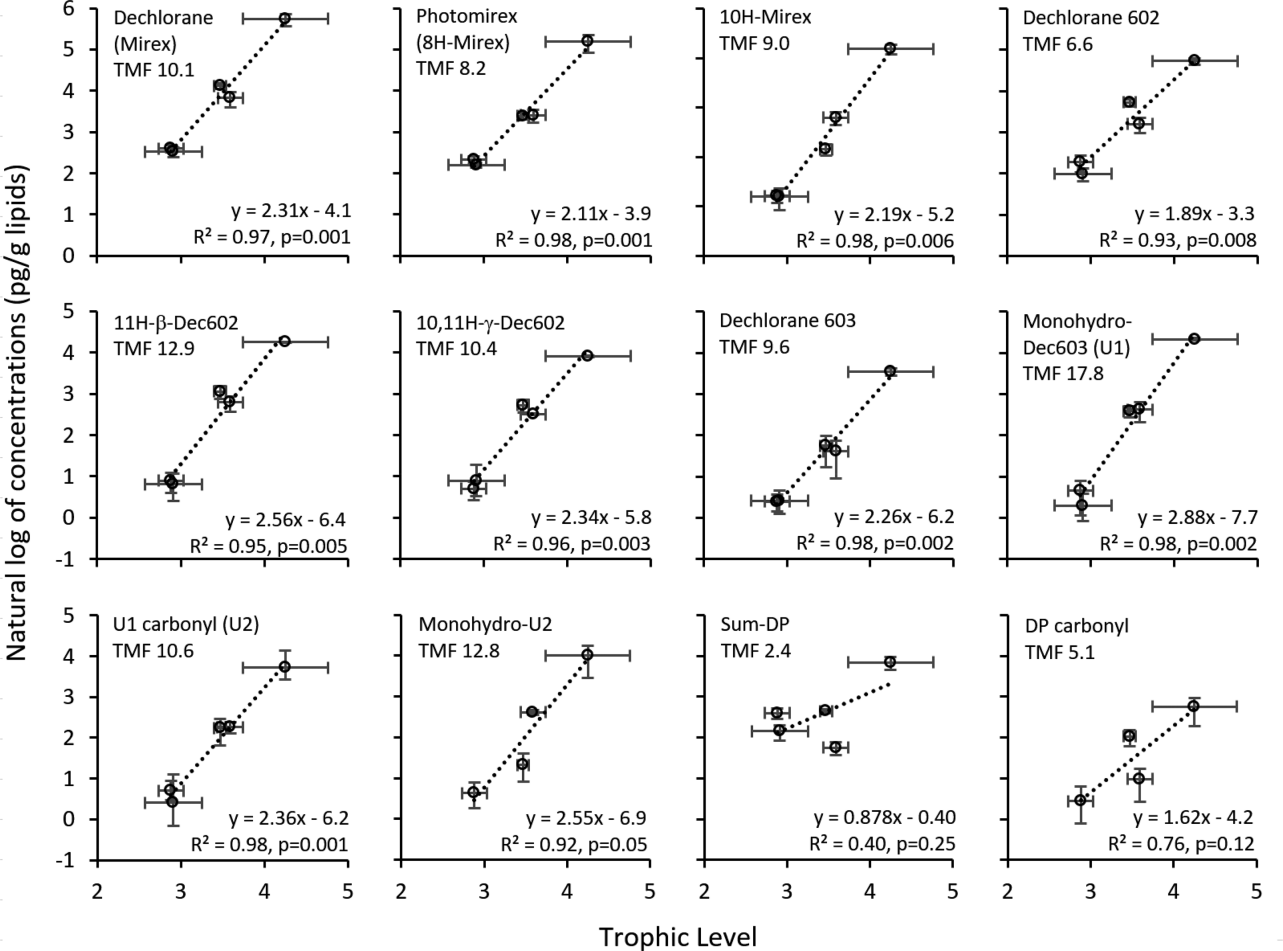
Chemical concentrations of Dechlorane (Mirex), Dechlorane 602 (Dec602), Dechlorane 603 (Dec603), *syn*/*anti*-Dechlorane Plus (Sum-DP), and their monohydro (+H–Cl) and carbonyl transformation products in organisms of the Baltic Sea marine food web (pg/g lipids, log transformed) versus trophic level (TL). Slopes of the linear regression lines were used to calculate tropic magnification factors (TMFs) in the food web. The error bars correspond to one standard deviation.

Sufficient data for TMF calculations were available for 13 compounds (Figure 2). Significant biomagnification (*p* < 0.05) was observed for all except DP and DP-carbonyl. The TMFs were relatively high, ∼10, and there was little difference in TMF between the parent compound and its TPs (usually within a factor of 2), which indicated limited contributions from metabolic processes in the investigated species. This suggests that they are formed through biotic or abiotic processes prior to uptake. It has long been known that Mirex is reduced to photomirex and 10H-mirex and oxidized to Kepone (carbonyl of Mirex) upon irradiation with UV light.^22^ More recently, it has been reported that hydro-DPs are present as impurities in technical DP^15^ but also formed by UV degradation and microbial (anaerobic) degradation of DP.^23,24^ Dechloranes 602 and 603 have similar structures to Mirex and DP (highly chlorinated nonaromatic polycyclic hydrocarbons; Figure S2) and are, thus, expected to undergo similar transformation reactions in the environment.

The potential relationship between lipophilicity (LogP) and TMFs was investigated using LogP values estimated using the XLOGP3 model,^25^ selected because it yielded a value (5.3) close to the empirically determined LogP of Mirex (5.28).^26^ There was no strong correlation between LogP and TMFs (Figure S3).

There have been few biomagnification studies of dechloranes and related compounds, and only five have reported statistically significant TMFs.^27−31^ Most of the previously reported TMFs are lower than those reported here, although the value obtained for Mirex (10.1) falls between the two previously reported values (1.9 and 13).^27,28^ For Dec602 and Dec603, the values reported here are higher (6.6 vs. 3.7)^27^ and much higher (12.4 vs. 1.3),^28^ respectively, than previously reported values. For *syn*-DP and *anti*-DP, the previously reported values range from −0.79 to 2.9,^27,30,31^ and from 0.59 to 3.3,^27,29−31^ respectively. The corresponding (nonsignificant) values reported here were 2.5 and 2.4, which are within ranges of the cited values.

### Potential Importance of Results for POP Candidate Assessment

DP is currently under review for listing under the Stockholm Convention on POPs.^32^ Results obtained in this study suggest that additional dechloranes (besides Mirex and DP) and dechlorane-related compounds must be considered. DPs only account for a small fraction of the sum of dechloranes and dechlorane-related compounds (excluding Mirex, which is already listed) found in top predators (0.8% in porpoise, 2.1% in eagle, 3.8% in guillemot). Further, some dechlorane TPs occur at higher concentrations than the parent compound (e.g., Dechlorane 603 TPs were >10-fold more abundant than their parent), and concentrations of individual dechloranes in the most contaminated species (eagle) have changed little during the period 1965–2017 (median −1.8% per year; range −3.8% to +1.3% per year). Finally, we obtained clear evidence of biomagnification of all TPs of Mirex, Dec602, and Dec603, with TMFs (8.2 to 17.8) similar to those of the parent compounds (6.6 to 12.4), and much higher than that of DP (2.4, nonsignificant). Thus, there seem to be several reasons to expand the list of dechloranes considered for listing beyond the *cis*- and *trans*-isomers of DP.

## Supporting Information Available

Sample information; cleanup and GC-HRMS method information; detected compound names, molecular formulae, GC retention information, quantification ions and mass accuracies, and distributions between Florisil fractions; chemical structures of identified and tentatively identified compounds; HRMS spectra of tentatively identified compounds (PDF)
