# Supplementary material for: Biomagnification and Temporal Trends of New and Emerging Dechloranes and Related Transformation Products in Baltic Sea Biota
Source: Environ Sci Technol Lett. 2022 Apr 13;9(5):406–12. doi: 10.1021/acs.estlett.2c00171 (PMC9097483; doi:10.1021/acs.estlett.2c00171)
Supplement: Supplementary file 1 — ez2c00171_si_001.pdf [file ez2c00171_si_001.pdf]

Biomagnification and Temporal Trends of New and Emerging Dechloranes and Related Transformation Products in Baltic Sea Biota

Peter Haglund, Andriy Rebryk

Table S1. Species, Tissues and Dates of Investigated Baltic Sea Fish, Bird and Harbor Porpoise Samples (*n*= Number of Subsamples in Pool), with Sample Location Coordinates According to WGS 84 and Masses (Wet Weight, Except for Guillemot, for which Previously Extracted Lipids were Analyzed).

Species	Tissue	Year	Location	N	E	*n*=	Mass (g)	Lipids (%)
Eelpout	Muscle	1995	Kvädöfjärden	64.34725	61.0472	10	49.3	1.0
Eelpout	Muscle	1998	Kvädöfjärden	64.34725	61.0472	10	49.2	1.4
Eelpout	Muscle	2001	Kvädöfjärden	64.34725	61.0472	10	49.6	1.2
Eelpout	Muscle	2004	Kvädöfjärden	64.34725	61.0472	10	49.5	1.0
Eelpout	Muscle	2006	Kvädöfjärden	64.34725	61.0472	10	49.4	0.81
Eelpout	Muscle	2009	Kvädöfjärden	64.34725	61.0472	10	49.4	0.92
Eelpout	Muscle	2012	Kvädöfjärden	64.34725	61.0472	10	49.5	1.2
Eelpout	Muscle	2014	Kvädöfjärden	64.34725	61.0472	10	49.3	1.0
Eelpout	Muscle	2017	Kvädöfjärden	64.34725	61.0472	10	49.0	1.0
Herring	Muscle	1986	Landsort	65.11972	66.4086	10	29.9	5.0
Herring	Muscle	1987	Landsort	65.11972	66.4086	10	29.8	3.7
Herring	Muscle	1990	Landsort	65.11972	66.4086	10	28.9	3.0
Herring	Muscle	1994	Landsort	65.11972	66.4086	10	30.1	5.0
Herring	Muscle	1996	Landsort	65.11972	66.4086	10	29.6	2.1
Herring	Muscle	1999	Landsort	65.11972	66.4086	10	29.9	4.1
Herring	Muscle	2001	Landsort	65.11972	66.4086	10	29.9	4.1
Herring	Muscle	2004	Landsort	65.11972	66.4086	10	29.8	1.8
Herring	Muscle	2009	Landsort	65.11972	66.4086	10	29.8	5.0
Herring	Muscle	2014	Landsort	65.11972	66.4086	10	29.9	4.3
Herring	Muscle	2017	Landsort	65.11972	66.4086	10	29.9	4.0
Herring	Muscle	2018	Landsort	65.11972	66.4086	10	28.8	3.3
Guillemot	Egg*	1986	Stora Karlsö	63.52833	67.9264	5	0.40*	100*
Guillemot	Egg	1989	Stora Karlsö	63.52833	67.9264	5	0.50	100
Guillemot	Egg	1992	Stora Karlsö	63.52833	67.9264	10	0.40	100
Guillemot	Egg	1995	Stora Karlsö	63.52833	67.9264	5	0.50	100
Guillemot	Egg	1998	Stora Karlsö	63.52833	67.9264	5	0.40	100
Guillemot	Egg	2001	Stora Karlsö	63.52833	67.9264	5	0.40	100
Guillemot	Egg	2004	Stora Karlsö	63.52833	67.9264	5	0.30	100
Guillemot	Egg	2007	Stora Karlsö	63.52833	67.9264	5	0.40	100
Guillemot	Egg	2010	Stora Karlsö	63.52833	67.9264	5	0.40	100
Guillemot	Egg	2013	Stora Karlsö	63.52833	67.9264	5	0.40	100
Guillemot	Egg	2018	Stora Karlsö	63.52833	67.9264	5	0.30	100
Guillemot	Egg	2019	Stora Karlsö	63.52833	67.9264	5	1.4	100
Porpoise	Blubber	1988	SW Baltic Proper	Multiple locations		3	9.3	90.2
Porpoise	Blubber	1991	SW Baltic Proper	Multiple locations		3	9.4	91.7
Porpoise	Blubber	1996	SW Baltic Proper	Multiple locations		3	9.4	93.4
Porpoise	Blubber	2001	SW Baltic Proper	Multiple locations		1	9.3	92.5
Porpoise	Blubber	2004	SW Baltic Proper	Multiple locations		1	9.2	90.6
Porpoise	Blubber	2008	SW Baltic Proper	Multiple locations		4	9.3	94.3
Porpoise	Blubber	2012	SW Baltic Proper	Multiple locations		3	9.2	92.9
Porpoise	Blubber	2016	SW Baltic Proper	Multiple locations		2	9.3	91.2
Porpoise	Blubber	2019	SW Baltic Proper	Multiple locations		2	9.2	92.7
White-tailed sea eagle	Muscle	1965	Swedish Baltic Proper Coast	Multiple locations		1	0.38	7.8
White-tailed sea eagle	Muscle	1983/84**	Swedish Baltic Proper Coast	Multiple locations		5	12.3	11.8
White-tailed sea eagle	Muscle	1991	Swedish Baltic Proper Coast	Multiple locations		5	8.0	7.1
White-tailed sea eagle	Muscle	1996	Swedish Baltic Proper Coast	Multiple locations		5	11.1	7.7
White-tailed sea eagle	Muscle	2001	Swedish Baltic Proper Coast	Multiple locations		7	19.7	4.9
White-tailed sea eagle	Muscle	2006	Swedish Baltic Proper Coast	Multiple locations		11	41.6	7.5
White-tailed sea eagle	Muscle	2011	Swedish Baltic Proper Coast	Multiple locations		10	36.9	8.0
White-tailed sea eagle	Muscle	2017	Swedish Baltic Proper Coast	Multiple locations		10	18.6	8.1

* Guillemot egg samples were provided in the form of extracted fat. No lipid content information is therefore available for individual samples, but the average fat content of guillemot eggs from the area and time period is 12%.

** Samples for two sampling years were pooled together.

Table S2. Equipment, Solvents and Settings Used in the Clean-up of Dechloranes and Dechlorane-Related Compounds by High-Resolution Gel Permeation Chromatography (HR-GPC) and Florisil® Column Chromatography.

Clean-up	Specification	Additional information
HR-GPC		
HPLC	Agilent 1260	Agilent, Santa Clara, CA, USA
Column #1	5µm PL gel, 7.5 × 300 mm, 100Å	Agilent, Santa Clara, CA, USA
Column #2	5µm PL gel, 7.5 × 300 mm, 50 Å	Agilent, Santa Clara, CA, USA
Eluent	Dichloromethane:n-hexane, 1:1	SupraSolv®; Merck, Darmstadt, Germany
Injection volume	900 µL	
Flow	1 mL/min	
Waste fraction	0-15 min	
Contaminant fraction	15-40 min	
Calibrant	Corn oil	Fraction change at end of corn oil peak
Florisil®		
Column	Open column, 10 mm i.d.	
Adsorbent	8 g of 1.2% water deactivated Florisil®	Florisil® for column chromatography; Merck, Germany
Fraction 1	38 mL n-hexane	SupraSolv®; Merck, Darmstadt, Germany
Fraction 2	34 mL 15% dichloromethane in n-hexane	SupraSolv®; Merck, Darmstadt, Germany
Fraction 3	54 mL 50% dichloromethane in n-hexane	SupraSolv®; Merck, Darmstadt, Germany
Fraction 4	80 mL 8% methanol in dichloromethane	SupraSolv®; Merck, Darmstadt, Germany

Table S3. Retention Times (RT), Linear Retention Indices (LRIs), Molecular Formulae, Quantification Ions (Quan m/z), Mass Accuracies (ppm; experimental mass – theoretical mass), and Distributions Between Florisil® Fractions One, Two and Three of Analytes.

Name	RT (min)	LRI	Formula	Quan m/z	ppm	Fr 1	Fr 2	Fr 3
Dechlorane (Mirex)	41.92	2546	C10Cl12	271.8096*	1.1	99%	1%	0%
Photomirex (8H-mirex)	38.78	2393	C10HCl11	271.8096*	0.4	99%	1%	0%
10H-mirex	39.73	2438	C10HCl11	271.8096*	-1.5	100%	0%	0%
Dechlorane 602	45.34	2728	C14H4Cl12O	613.6441	1.1	34%	66%	0%
Dechlorane 602, isomer #1	47.16	2831	C14H4Cl12O	613.6441	−2.0	2%	98%	0%
Dechlorane 602, isomer #2	48.35	2902	C14H4Cl12O	613.6441	1.8	0%	100%	0%
Monohydro Dechlorane 602 #1	43.88	2648	C14H5Cl11O	577.6861	−2.8	0%	100%	0%
Monohydro Dechlorane 602 #2	43.99	2654	C14H5Cl11O	577.6861	0.3	4%	96%	0%
11H-α-Dechlorane 602	44.28	2670	C14H5Cl11O	577.6861	4.3	5%	95%	0%
Monohydro Dechlorane 602 #4	44.82	2699	C14H5Cl11O	577.6861	2.3	0%	100%	0%
11H-β-Dechlorane 602	45.02	2710	C14H5Cl11O	577.6861	3.1	1%	99%	1%
10,11-dihydro-Dechlorane 602 (α)	43.05	2604	C14H6Cl10O	543.7250	−4.4	0%	100%	0%
10,11-dihydro-Dechlorane 602 (γ)	44.53	2683	C14H6Cl10O	543.7250	−0.6	0%	85%	15%
Dechlorane 603	52.94	3198	C17H8Cl12	637.6805	0	12%	88%	0%
Dechlorane 603, isomer	57.26	3514	C17H8Cl12	637.6805	1.4	0%	71%	29%
Monohydro Dechlorane 603 (U1)	53.88	3263	C17H9Cl11	601.7225	2.0	0%	99%	1%
Dihydro Dechlorane 603	53.56	3241	C17H10Cl10	567.7614	−3.0	0%	44%	56%
Monohydro Dechlorane 603, carbonyl- (U2)	53.93	3267	C17H7Cl11O	615.7017	−1.9	0%	0%	100%
Dihydro Dechlorane 603, carbonyl- #1	52.53	3170	C17H8Cl10O	581.7407	−0.3	0%	0%	100%
Dihydro Dechlorane 603, carbonyl- #2	52.90	3195	C17H8Cl10O	581.7407	−0.3	0%	4%	96%
Dihydro Dechlorane 603, carbonyl- #3	53.27	3220	C17H8Cl10O	581.7407	−2.9	0%	0%	100%
Trihydro Dechlorane 603, dicarboxy- #1	51.87	3125	C17H9Cl9O2	563.7746	0.7	0%	5%	95%
Trihydro Dechlorane 603, dicarboxy- #2	52.54	3170	C17H9Cl9O2	563.7746	−3.9	0%	2%	98%
Monohydro Dechlorane 603, hydroxy-	57.10	3501	C17H9Cl11O	617.7174	−4.9	0%	0%	100%
Dechlorane Plus, syn-	57.75	3552	C18H12Cl12	653.7118	2.6	13%	87%	0%
Dechlorane Plus, anti-	58.94	3648	C18H12Cl12	653.7118	3.1	11%	89%	0%
Monohydro Dechlorane Plus, syn-	56.42	3449	C18H13Cl11	619.7508	3.9	0%	100%	0%
Monohydro Dechlorane Plus, anti-	58.35	3600	C18H13Cl11	619.7508	4.5	0%	100%	0%
Dihydro Dechlorane Plus, anti-	54.89	3336	C18H14Cl10	583.7927	−1.4	0%	100%	0%
Dechlorane Plus, carbonyl- #1	58.24	3591	C18H10Cl12O	667.6911	−3.6	0%	0%	100%
Dechlorane Plus, carbonyl- #2	59.67	3708	C18H10Cl12O	667.6911	1.3	0%	0%	100%

* Compounds analyzed using electron ionization (EI). All other compounds analyzed using electron capture negative ion chemical ionization (ECNI).

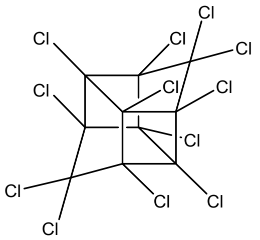

Mirex

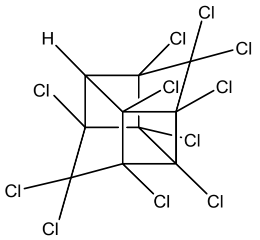

8H-Mirex

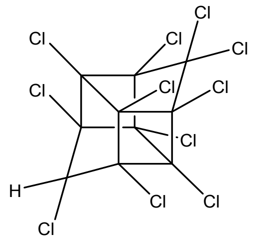

10H-Mirex

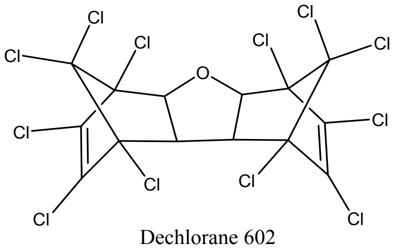

Dechlorane 602

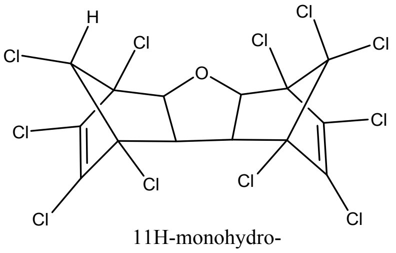

11H-monohydro-
Dechlorane 602

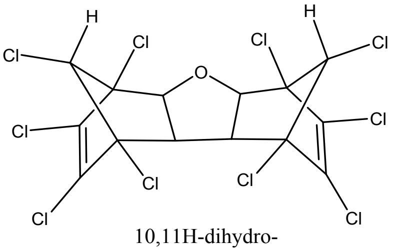

10,11H-dihydro-
Dechlorane 602

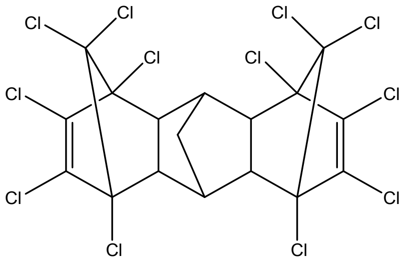

Dechlorane 603

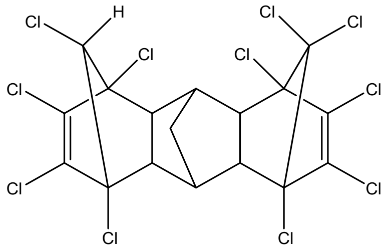

Monohydro-
Dechlorane 603 (U1)

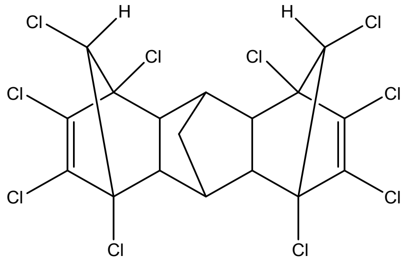

Dihydro-
Dechlorane 603

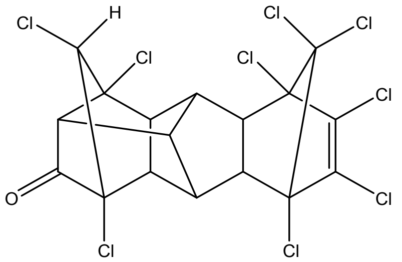

Monohydro-
Dechlorane 603,
carbonyl (U2)

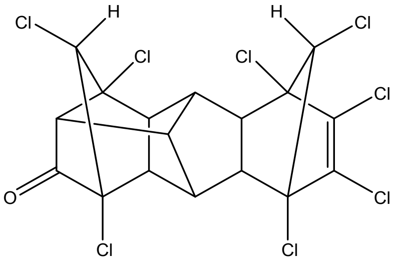

Dihydro-
Dechlorane 603, carbonyl-
(monohydro U2)

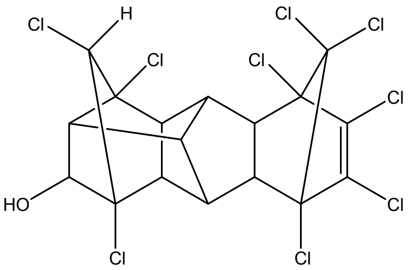

Monohydro-
Dechlorane 603, hydroxy-

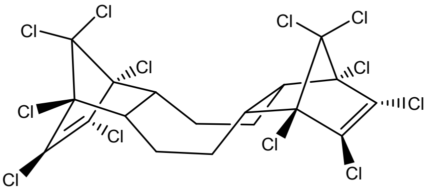

syn-Dechlorane Plus

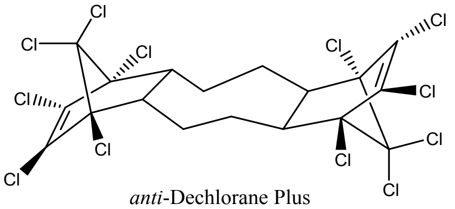

anti-Dechlorane Plus

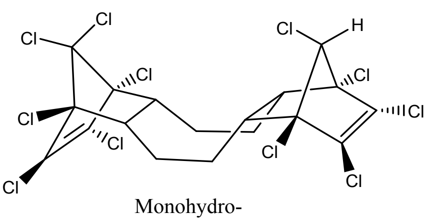

Monohydro-
syn-Dechlorane Plus

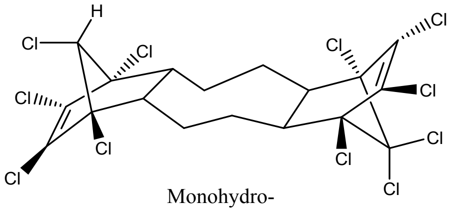

Monohydro-
anti-Dechlorane Plus

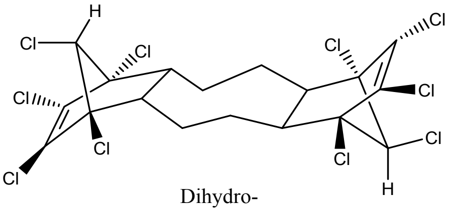

Dihydro-
anti-Dechlorane Plus

Figure S1. Proposed structures of detected compounds. Structures of hydro-mirex were assigned using (S1, S2), hydro-Dec602 using (S3), hydro-Dechlorane Plus using (S4, S5) and U1, U2, and hydroxyl-Dec603 using (S6). The stereochemistry of the dihydro congeners of Dechlorane 602, 603 and Plus are uncertain.

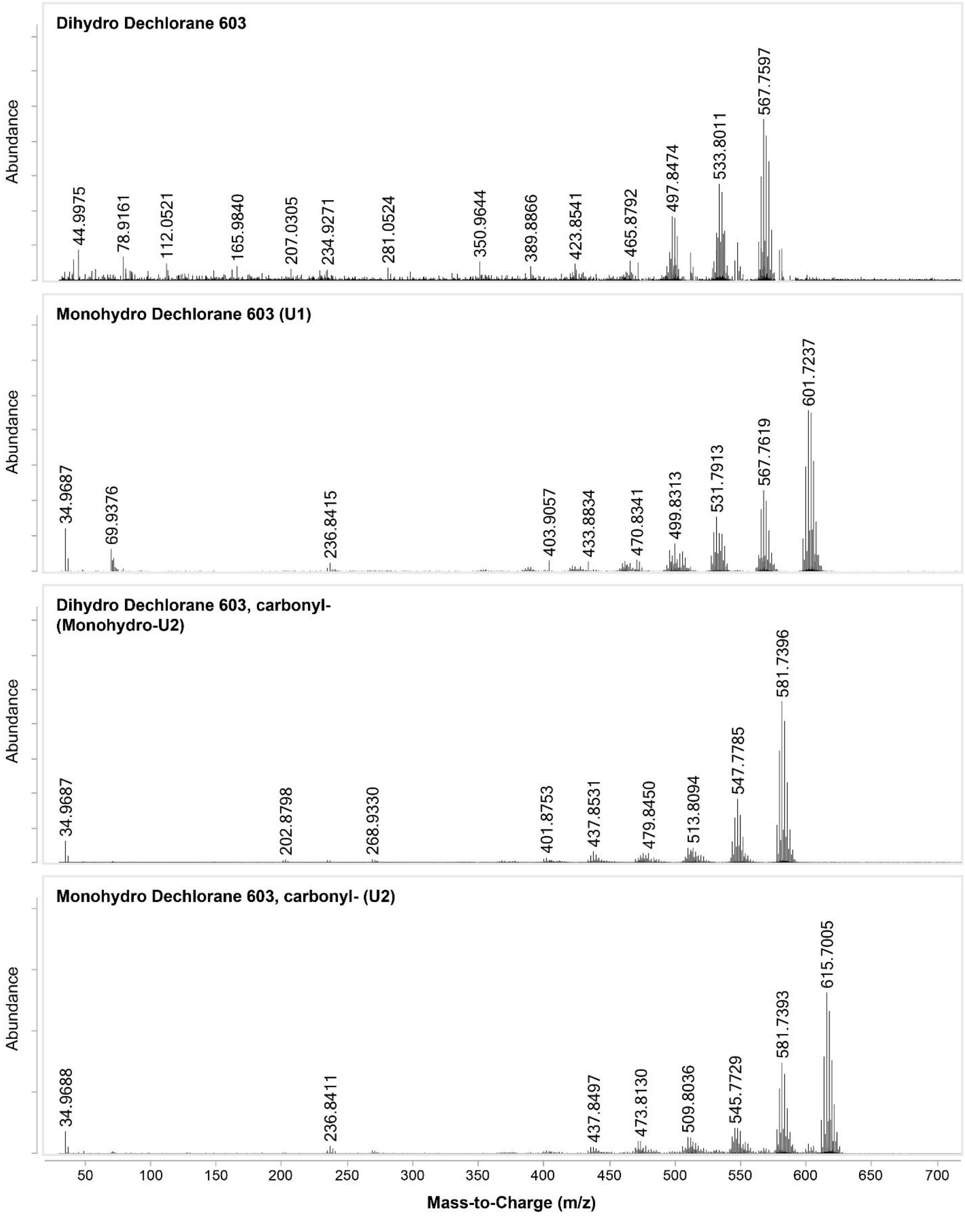

Figure S2. Spectra of tentatively identified new and emerging Dechlorane 603 and Dechlorane Plus transformation products. U1 and U2 refer to a monohydro-Dechlorane 603 isomer and its carbonyl oxidation product, identified by Liu et al. 2019 (*Environ. Sci. Technol.* 2019, *53*, 3419-3428).

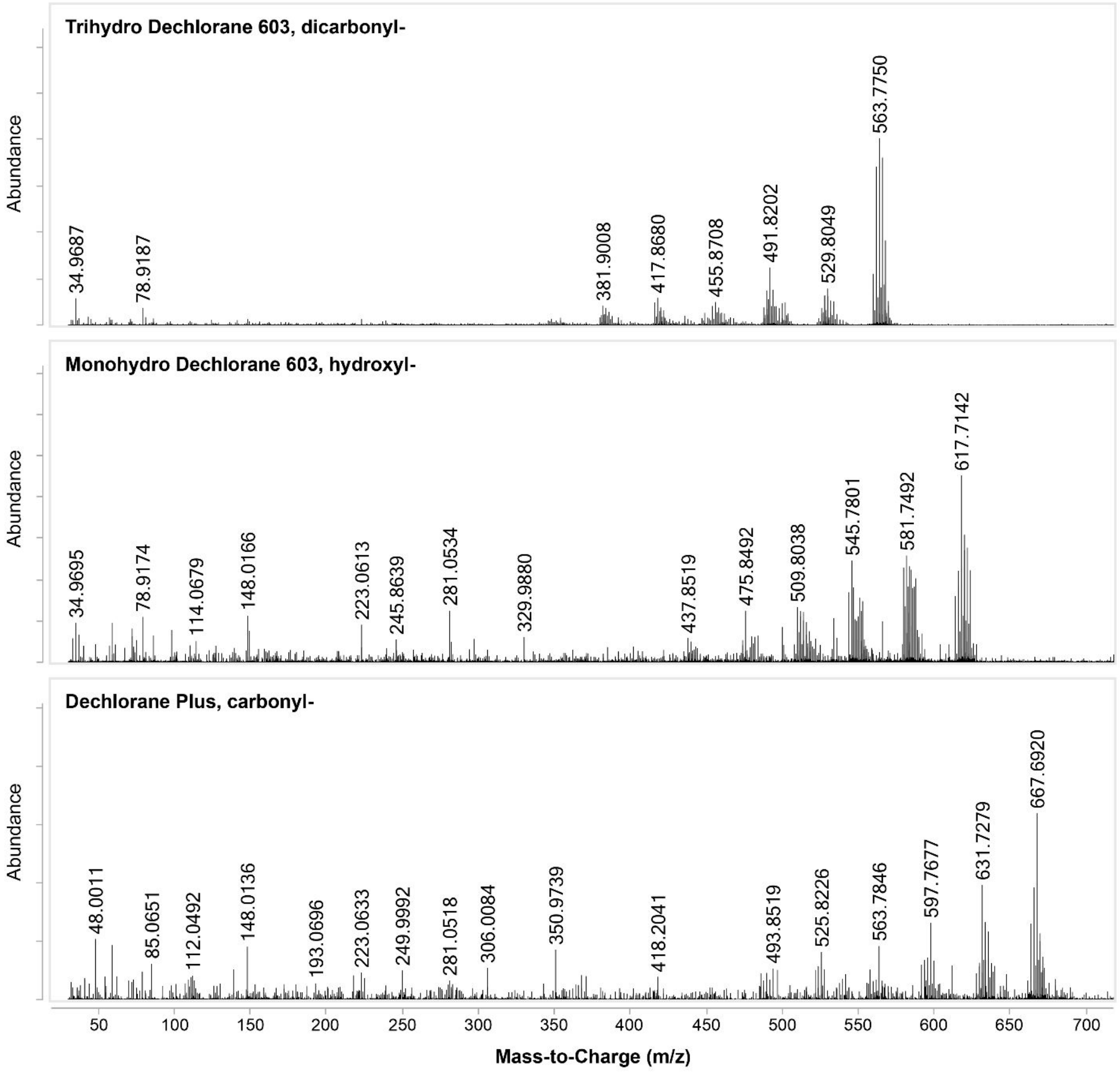

Figure S2, continued. Spectra of tentatively identified new and emerging Dechlorane 603 and Dechlorane Plus transformation products.

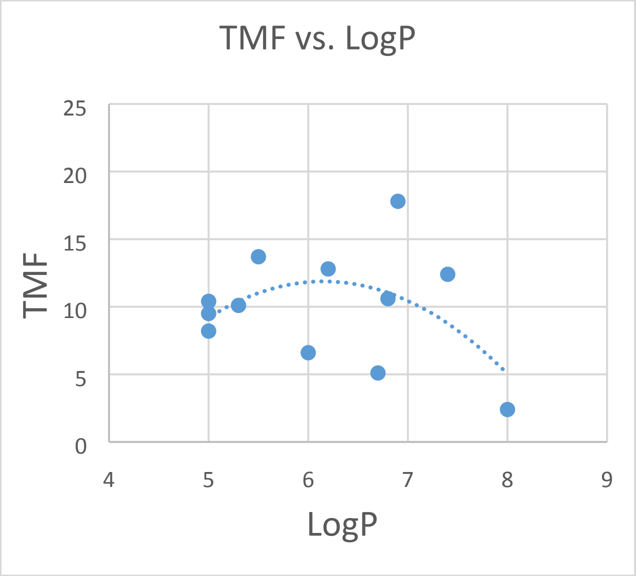

Figure S3. Graph of the trophic magnification factors (TMFs) of dechloranes and dechlorane transformation products and the LogP values, calculated using XLOGP3. The line represents the best-fit function to the data.

References

(S1) Norstrom, R. J.; Hallett, D. J.; Onuska, F. I.; Comba, M. E. Mirex and its degradation products in Great Lakes herring gulls. Environ. Sci. Technol. 1980, 14, 860-866.

(S2) Carlson, D. L.; Konyha, K. D.; Wheeler, W. B.; Marshall, G. P.; Zaylskie, R. G. Mirex in the Environment: Its Degradation to Kepone and Related Compounds. *Science* 1976, *194*, 939-941.

(S3) Shen, L.; Jobst, K. J.; Helm, P. A.; Reiner, E. J.; McCrindle, R.; Tomy, G. T.; Backus, S.; Brindle, I. D.; Marvin, C. H. Identification and determination of the dechlorination products of Dechlorane 602 in Great Lakes fish and Arctic beluga whales by gas chromatography–high resolution mass spectrometry. *Anal. Bioanal. Chem.* 2012, *404*, 2737–2748.

(S4) Brazeau, A.L.; Pena-Abaurrea, M.; Shen, L.; Riddell, N.; Reiner, E.J.; Lough, A. J; McCrindle, R.; Chittim, B. Dechlorinated Analogues of Dechlorane Plus. *Environ. Sci. Technol.* 2018, 52, 5619−5624

(S5) Sverko, E.; McCarry, B.; McCrindle, R.; Brazeau, A.; Pena-Abaurrea, M.; Reiner, E.; Smyth, S. E.; Gill, B.; Tomy, G. T. Evidence for Anaerobic Dechlorination of Dechlorane Plus in Sewage Sludge. *Environ. Sci. Technol.* 2015, *49*, 13862-13867.

(S6) Liu, X.; Wu, Y.; Zhang, X.; Shen, L.; Brazeau, A. L.; Adams, D.H.; Marler, H.; Watts, B. D.; Chen, D. Novel dechlorane analogues and possible sources in peregrine falcon eggs and shark livers from the Western North Atlantic regions. *Environ. Sci. Technol.* 2019, *53*, 3419−3428.
